# Ensuring continuity of malaria services in times of crisis: lessons learned in Uganda during the COVID-19 pandemic

**DOI:** 10.1093/inthealth/ihaf107

**Published:** 2025-10-28

**Authors:** Emma K Manning, Godfrey Magumba, James K Tibenderana, Anthony Nuwa

**Affiliations:** Malaria Consortium, 244-254 Cambridge Heath Road, London E2 9DA, UK; Malaria Consortium, Plot 25, Upper Naguru East Road, Kampala, Uganda; Malaria Consortium, 244-254 Cambridge Heath Road, London E2 9DA, UK; Malaria Consortium, Plot 25, Upper Naguru East Road, Kampala, Uganda

**Keywords:** COVID-19, malaria, pandemic response, seasonal malaria chemoprevention, Uganda

## Abstract

During the coronavirus disease 2019 pandemic, Uganda’s National Malaria Control Division (NMCD) worked with partners to sustain essential malaria control interventions despite the constraints of national pandemic guidelines. To continue delivering programs, the NMCD rapidly introduced updated operational protocols. In parallel, partners secured funding to supply >33 000 health workers and village health teams with infection prevention and control equipment, ensuring safe delivery of services. As a long-standing partner of the NMCD, Malaria Consortium provided critical support throughout the pandemic. This case study captures the key insights gained from maintaining malaria services during a public health crisis, highlighting the importance of proactive risk planning, timely data reporting and sustainable sources of funding and commodities.

## Introduction

Uganda has made steady progress against malaria, reducing incidence by >50% and mortality by >20% between 2000 and 2019.^1^ However, the disease burden remains high, with the country accounting for 5% of cases and 3% of deaths from malaria globally. At the onset of the coronavirus disease 2019 (COVID-19) pandemic in March 2020, several districts of Uganda were already experiencing increased malaria transmission. The pandemic placed additional pressure on the health system, complicating the efforts of the Ministry of Health (MOH) to maintain essential services. As a long-standing partner, Malaria Consortium was well established across the country, enabling us to support the MOH in sustaining malaria interventions and strengthening infection and prevention (IPC) measures. This case study documents lessons learned from operating during the pandemic and reflects on how they can be applied to strengthen malaria programming in Uganda against emerging threats.

## COVID-19 disruptions

At the onset of the COVID-19 pandemic, the novelty of the disease meant that global guidelines were being developed rapidly as information emerged. Once available, these guidelines were primarily based on non-African contexts and largely relied on symptomatic case isolation, indicating that anyone with a fever should be quarantined until they had received a negative test for COVID-19. However, due to resource and capacity constraints, diagnostic results took 3 weeks to confirm in Uganda and isolation facilities were limited, making this guidance impractical. Furthermore, the symptoms of COVID-19 overlapped with those of endemic diseases like malaria, complicating case management of all common illnesses. Health workers were afraid to treat anyone with a fever for fear that it was COVID-19. Moreover, the general public was afraid to seek treatment for fever out of fear of stigma or being placed in isolation.

Beyond behavioural changes, health services were further disrupted by lockdowns and social distancing, which restricted movement and limited public transportation, hindering access. Due to the global impact of the pandemic, many donors reduced funding or closed programs to focus on their own economies and manufacturers pivoted their resources to focus on COVID-19, resulting in shortages of malaria commodities.

## Uganda’s pandemic response

### Adapting guidelines to country context

As chair of the malaria case management technical working group, our staff collaborated with the MOH and other partners to develop new protocols for COVID-19 case management in Uganda. Due to the high prevalence of malaria, which requires prompt treatment, we advocated for all patients presenting with a fever to be tested for malaria in the first instance before being presumed to have COVID-19.

Guidelines for malaria case management also had to be adapted due to a global shortage of malaria rapid diagnostic tests (mRDTs), which resulted from manufacturers pivoting their focus to producing rapid diagnostic tests for COVID-19. Consequently, stock-outs of mRDTs hindered adherence to case management guidelines, which require a positive malaria test prior to initiating treatment. When diagnostic tests were not available, we worked with the affected communities to provide them with alternative guidelines. Health workers were advised to treat anyone presenting with a fever for malaria in the first instance and, if they did not respond to treatment within 2 d, to then consider alternative conditions.

### Reducing fear

To reduce fear among health workers, and protect them from contracting COVID-19, additional funding was needed to procure and implement IPC measures for frontline workers. We received funding from the Mastercard Foundation to train a quarter of Uganda’s village health workers on IPC and to procure personal protective equipment (PPE) for them including masks, goggles, soap and sanitisers. With support from the MOH, we delivered this equipment to 40 districts, protecting 33 000 health providers, including village health teams (VHTs) and health facility workers. Community outreach was also essential, prompting us to disseminate health communications through radio messages that encouraged people to seek treatment promptly if they had a fever.

### Adapting program delivery

To ensure malaria services were able to continue, some standard operating procedures had to be adapted to adhere to COVID-19 guidelines. Long-lasting treated net (LLIN) campaigns could no longer be delivered through a fixed-point delivery strategy in which households collect their nets from a centralised distribution point, as large gatherings were prohibited. A door-to-door distribution campaign was devised to avoid bringing groups of people together, which allowed campaigns to go ahead as planned despite some delays.

In addition, due to lockdowns and restrictions on movement, our staff were unable to provide in-person support to health workers. Instead, remote and phone-based systems were set up to ensure that support and supervision were sustained to ensure programs were properly implemented. Health centres were also supported to rearrange their facilities to allow for social distancing, including offering some services outside or under tents.

For seasonal malaria chemoprevention (SMC) delivery, we encouraged the VHTs distributing SMC to maintain social distancing while administering the sulfadoxine-pyrimethamine with amodiaquine (SPAQ) treatment to eligible children. For example, caregivers were guided by the VHTs at a distance, outside of the house, to administer the medicines to the children. All research assistants carrying out data collection for SMC were tested for COVID-19 to ensure they were not infected. This was in addition to standard COVID-19 safety measures such as social distancing and maintaining good hand hygiene while interacting with households.

On top of these program adaptations, Malaria Consortium conducted a study on COVID-19 and malaria to better understand the relationship between the two diseases to improve planning for malaria interventions.^2^

## Looking forward: risk planning and system strengthening

Through sustained collaboration between the MOH and partners, malaria service delivery remained largely uninterrupted during the COVID-19 pandemic, despite significant operational challenges. However, retrospective analysis has revealed several opportunities to enhance system resilience and improve the timeliness and coordination of responses to future public health emergencies. Below we have identified areas for strengthening Uganda’s pandemic preparedness and response, which have been shared with the Ugandan NMCD.

### Update the epidemic response plan for malaria jointly with all sectors and partners to develop crosscutting guidelines

Despite Uganda’s experience with epidemics like malaria and Ebola, there was lack of a crosscutting national disease outbreak preparedness and response guidelines at the outset of the COVID-19 pandemic. While these documents were being updated and revised, partner organisations were working independently to adapt their programs, which led to an uncoordinated response. This gap between disease emergence and a coordinated response showed the importance of up-to-date national and subnational epidemic response plans to ensure that the country and districts have resources when, and where, they are needed during emergency scenarios.

### Invest in sustainable domestic programs

External funding sources can be unreliable during a crisis, as was evident from the premature closure of the UK’s Foreign, Commonwealth and Development Office (FCDO)-funded ‘Strengthening Uganda’s response to malaria’ (SURMa) project and shortages of mRDTs from global manufacturers. The closure of this program and stock-outs of mRDTs hindered the activities of malaria programs across the country, highlighting the need for investment in sustainable domestic programs.

### Establish policies to safeguard the supply of malaria commodities during epidemics and promote local manufacturing to improve commodity security

Rapid adaptation of protocols and strong communication between organisations were essential for the success of malaria programs during the COVID-19 pandemic. However, supply chain networks were vulnerable based on their reliance on external partners and gaps in guidelines. Crosscutting policies are needed for disease outbreak response that engage all sectors and partners to merit buy-in. Awareness of these plans will enable a coordinated response between organisations when an epidemic that threatens the continuity of malaria services emerges. These policies should identify sources of emergency funding to procure commodities during epidemics but should also be supported by an adequate supply of PPE and malaria commodities to prevent stock-outs. The risk of stock-outs could also be reduced through promotion of local or regional manufacturing of quality-assured goods, thus increasing commodity security.

### Build resilience into manufacturing and supply chains

Policies are needed that will safeguard the supply of malaria commodities during epidemics. Local manufacturing would promote an overarching framework for national and regional commodity security, making manufacturing and supply chains more resilient to external threats.

### Invest in implementation and scale-up of digital tools used by VHTs to improve data quality and real-time reporting

During the pandemic, many areas of Uganda still relied on paper-based reporting systems in which VHTs physically submit reports each month to health facilities and data are then aggregated and sent to a district health team for input into an electronic health system. When travel restrictions disrupted these processes, preventing VHTs from travelling to health facilities, data reporting was delayed, which slowed response times. Digitalisation of data entry enables near real-time reporting across the country, reducing contact between teams and reducing travel time for VHTs. Malaria Consortium has piloted implementation and training of digitalised health programs in multiple districts across Uganda, but national coverage has not yet been achieved.^3^ Innovative programs that implementation and scale-up of digital tools used by VHTs would improve data quality and real-time reporting to support a robust health system.

## Data Availability

The data underlying this article will be shared upon reasonable request to the corresponding author.
